# Congenital Splenorenal Shunt with Characteristic Ultrasonographic Findings

**DOI:** 10.31662/jmaj.2018-0056

**Published:** 2019-02-01

**Authors:** Sho Kitagawa, Ai Minoura

**Affiliations:** 1 Department of Gastroenterology, Sapporo Kosei General Hospital, Sapporo, Japan

**Keywords:** splenorenal shunt, congenital, ultrasonography, color Doppler imaging

A 46-year-old woman presented for screening ultrasound examination. She had no history to suspect encephalopathy. During abdominal sonography, the direction of splenic venous flow was observed to be completely inverted in accordance with respiratory movements, namely, being hepatofugal in the inspiratory phase and hepatopetal in the expiratory phase (Figure 1). Subsequent computed tomography revealed a large tortuous vein between the splenic vein and the left renal vein (Figure 2). She had no history of trauma or surgery, and further examination showed no signs of portal hypertension. These findings were consistent with a diagnosis of congenital splenorenal shunt. Because her plasma ammonia levels were not elevated, the splenorenal shunt has been followed-up without any treatment. Congenital splenorenal shunt is a rare anomaly in patients without cirrhosis ^(1)^. In patients with portal hypertension, the direction of the splenic venous flow is to-and-fro or hepatofugal ^(2)^. In this case, the characteristic ultrasound findings were helpful for the diagnosis of splenorenal shunt.

**Figure 1.**
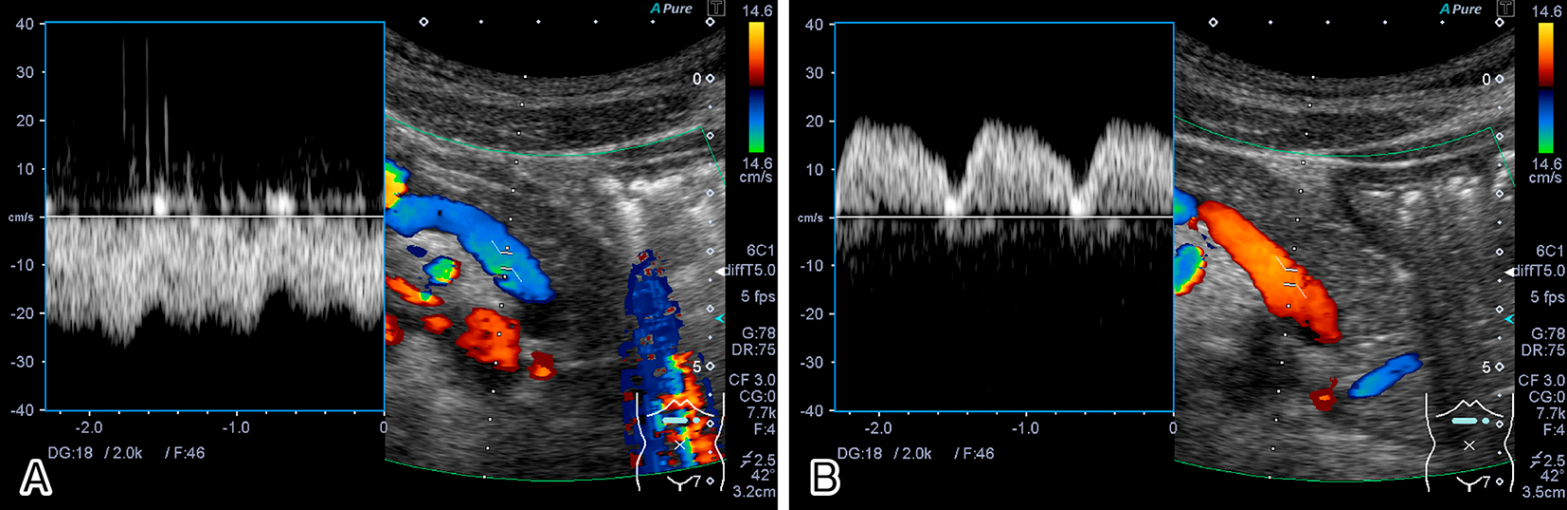
Color Doppler ultrasonographic images showing the splenic vein. (A) The splenic vein showed hepatofugal flow in the inspiratory phase. (B) The splenic vein showed hepatopetal flow in the expiratory phase.

**Figure 2.**
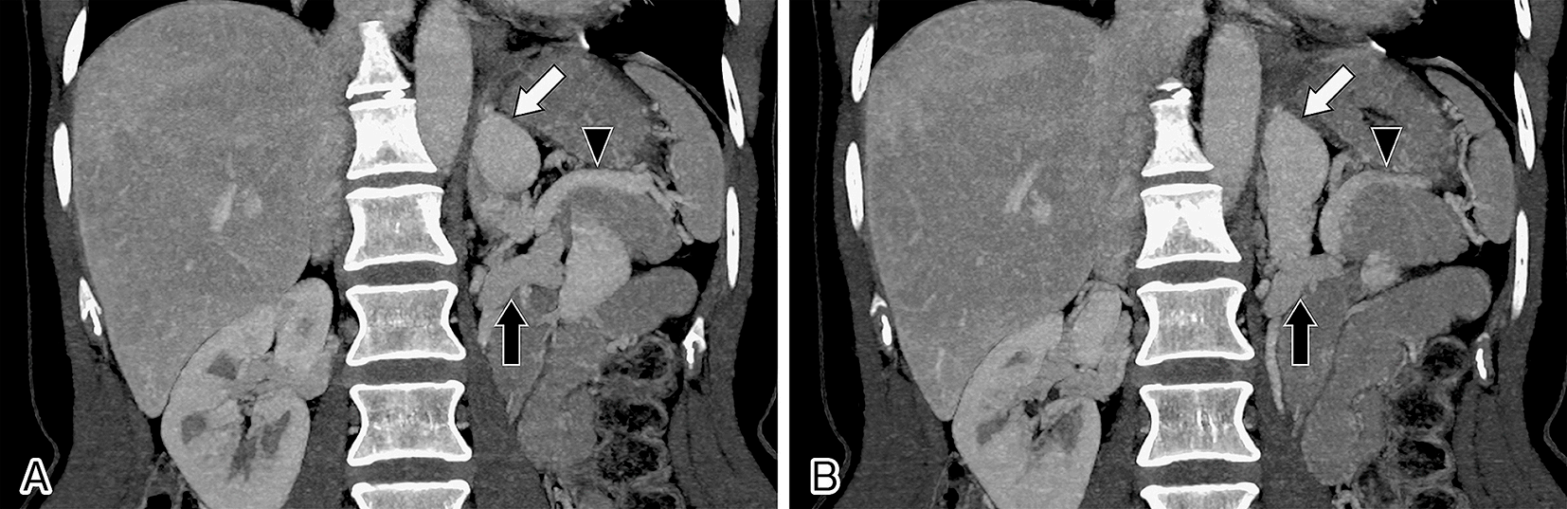
Computed tomographic scan images showing a splenorenal shunt (white arrows) between the splenic vein (black arrowheads) and the left renal vein (black arrows).

## Article Information

### Conflicts of Interest

None

### Author Contributions

SK wrote the manuscript. AM edited the manuscript.

### Informed Consent

We have obtained informed consent for this case report.
